# Pulsed electric field processing of edible insect slurries induces thermally-assisted microbial inactivation

**DOI:** 10.1016/j.crfs.2024.100940

**Published:** 2024-11-30

**Authors:** L.J.H. Sweers, M. Mishyna, L.M. Ahrné, R.M. Boom, V. Fogliano, T. Patra, C.M.M. Lakemond, J.K. Keppler

**Affiliations:** a Food Process Engineering Group, Wageningen University and Research, P.O. Box 17, 6700, AA Wageningen, the Netherlands; b Food Quality and Design Group, Wageningen University and Research, P.O. Box 17, 6700, AA Wageningen, the Netherlands; c Department of Food Science, University of Copenhagen, Rolighedsvej 30, DK-1958, Frederiksberg C, Denmark

**Keywords:** Lesser mealworm, House cricket, Mild processing, Microbial inactivation, Secondary protein structure

## Abstract

Insect-based food ingredients are emerging as sustainable protein sources, but their production requires ensuring microbial safety and inactivation of endogenous enzymes to avoid undesirable proteolysis, without compromising protein structure. While traditional thermal processing affects the protein structure, the potential of pulsed electric field (PEF) technology to inactivate microorganisms in lesser mealworm and house cricket slurries at pH 3 while simultaneously retaining the native protein structure is yet unexplored.

Lesser mealworm and house cricket slurries at pH 3 were subjected to continuous and batch PEF treatments with varying intensities (0–450 kJ/kg). Microbial inactivation (aerobes, anaerobes, yeasts, and moulds), temperature changes, protein solubility, protein structure (SDS-PAGE and FTIR), and endogenous protease activity were assessed.

For both insect species, high-intensity PEF (>150 kJ/kg) achieved up to 5 log microbial reduction, but increased temperatures up to 75 °C, altering protein structure. Low-intensity PEF did not affect protein conformation and protease activity, but was not effective in microbial inactivation (<1 log reduction).

We conclude that while PEF can effectively inactivate microorganisms, it cannot be considered a non-thermal method for the present sample conditions due to the temperature increase at higher intensities. PEF could be well-suitable for incorporation in hurdle techniques, such as combinations with moderate heating. Future research should investigate synergistic effects of PEF, also for using alternative PEF set-ups, with other mild processing techniques for effective microbial inactivation while preserving native protein structure. Furthermore, optimal PEF intensities for enhanced protein solubility should be explored.

## Highlights

• An effective PEF-induced microbial inactivation was coupled to a temperature increase.
• High-intensity PEF raised intermolecular β-sheets, likely causing protein aggregation.
• Low-intensity PEF did not affect protein structure and did not inactivate proteases.

## Graphical abstract

**Figure:**
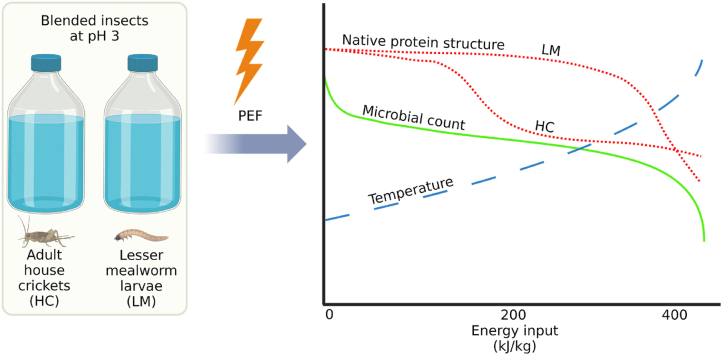

## 1 Introduction

Insects are one of the next-generation protein sources that may help answer the growing protein demand (Henchion et al., 2017). Insects are a more sustainable protein source compared to conventional meat sources, and insects provide proteins of excellent nutritional quality (Smetana et al., 2016; van Huis and Oonincx, 2017). Processing of insects is needed to increase consumer acceptability and also to inactivate microorganisms and endogenous enzymes that cause proteolysis and browning (Grabowski and Klein, 2017; Kröger et al., 2022; Yi et al., 2017). Currently, the insect industry often uses thermal processes such as blanching to achieve both microbial and enzyme inactivation. However, thermal treatments severely impair the functional properties (e.g. solubility) of insect proteins, which makes the incorporation of insect protein in high-quality food products challenging (Lee et al., 2019). To better retain the physicochemical and functional properties of the insect fractions while still achieving microbiological and enzymatic inactivation, milder processing techniques may be a solution. One of these milder techniques is pulsed electric field (PEF) processing.

PEF is a novel processing technique for microbial inactivation that is based on the concept of electroporation of cell membranes (Garner, 2019). Two or more electrodes apply a series of electrical pulses of high voltage within a short period. The electric field induces polarisation of the cell membrane, which in turn causes membrane poration (Toepfl et al., 2007). This electroporation allows mass transfer in and out of the cell. If the electroporation is sufficiently large, cells are permanently inactivated. PEF can also be used in combination with additional measures (e.g. antimicrobial compounds or moderate temperatures) as a hurdle technology (Zhao et al., 2012). Next to using PEF to induce microbial inactivation, PEF has been suggested for other applications, for example to support protein extraction from e.g. plant and microbial cells (Naliyadhara et al., 2022). The intensity and outcome of a PEF treatment are affected by many product and process factors, including the composition and conditions of the sample (e.g. conductivity), starting temperature, electric field strength, the number of pulses, and the pulse width (Taha et al., 2022).

Several studies have been performed on using PEF technology on insects. Most of these studies use PEF as a pre-treatment for more efficient drying of whole insects (Alles et al., 2020; Bogusz et al., 2022, 2023, 2024; El Hajj et al., 2023; Psarianos et al., 2024; Shorstkii et al., 2020). A few studies use PEF treatment to support protein, lipid, and chitin extraction in whole house crickets (*Acheta domesticus*) (Psarianos et al., 2022), yellow mealworms (*Tenebrio molitor*) (Smetana et al., 2020), and black soldier fly larvae (*Hermetia illucens*) (Alles et al., 2020). Limited knowledge is available on the effect of PEF specifically on insect proteins. Psarianos et al. (2022) found that PEF treatments (4.9–49.1 kJ/kg) can increase the emulsifying capacity (up to 75% increase compared to untreated), but did not affect the foaming capacity of house crickets.

None of the above studies focused on PEF-induced microbial inactivation of insect biomasses, although PEF has been successfully deployed for this purpose on various other foods (Garner, 2019; Zheng et al., 2024). We hypothesise that PEF could be a promising alternative to blanching for house cricket and lesser mealworm slurries because its effect is specifically targeted at microbial cell membranes and not at the proteins. In this framework, the effect of PEF treatment intensity on microbial decontamination and insect proteins should be assessed.

When using PEF for microbial decontamination, an acidic pH may be beneficial via the permeation of undissociated acids through the membrane, although this effect differs for different microorganisms (Ghoshal, 2023; Lima et al., 2023). Furthermore, previous studies found that an acidic pH of 3 ensures high protein solubility, avoids irreversible association of phenolics, and inhibits microbial growth, while it does not yet induce protein denaturation (Sweers et al., 2024b, Sweers et al., 2023).

Given the potential benefits of PEF technology and the advantages of processing at low pH, this study aims to investigate the efficacy of PEF in inactivating microorganisms in lesser mealworm and house cricket slurries at pH 3, while simultaneously preserving the native state of insect proteins. By combining PEF treatment with an acidic environment (i.e. pH 3), we hypothesise that effective microbial decontamination can be achieved while minimising alterations in protein structure. This study represents the first investigation of PEF-induced microbial inactivation in insect biomasses, while simultaneously investigating the effects of PEF treatment intensities on the insect proteins. This research thus aims to provide novel insights into developing more efficient and protein-friendly processing methods for the emerging insect-based food industry.

## 2 Materials and methods

### 2.1 Insects

House crickets (*Acheta domesticus*) and lesser mealworms (*Alphitobius diaperinus*) were purchased from Kreca Ento-Feed BV (The Netherlands). House crickets were supplied in the adult stage, lesser mealworms were supplied in the larval stage. All insects were starved for about 1 day at room temperature. After starvation, insects were frozen with liquid nitrogen and stored at −80 °C until further use.

### 2.2 Preparation of the slurry

Insects were mixed with Milli-Q water at a 1:5 (w/w) rate and subsequently ground for 1 min using a Thermomix blender (Vorwerk & Co. KG, Germany). The pH of the obtained slurry was set to 3 by using 5M HCl while stirring. The conductivities of the slurries at 35 °C were 4.4 mS/cm for house cricket slurry and 3.5 mS/cm for lesser mealworm slurry, as measured by a conductivity meter (Hach, U.S.).

### 2.3 Pulsed electric field (PEF) treatment

Insects were PEF-treated using a PEF Pilot Dual system (Elea GmbH, Germany). The electrodes generate monopolar pulses, and the pulse width was kept constant at 4 μs. Both a continuous and a batch set-up were used. There are several differences in the application of the technology in continuous and batch mode, such as the dimensions of the treatment cell and homogeneity. The conditions used for both set-ups are explained below.

#### 2.3.1 Continuous set-up

Insect slurries were PEF treated in a continuous flow system. The electrode gap was 1 cm. The electric field strength was varied (10/15/20 kV/cm). The slurry was passed through the system at 0.7 L/min using a peristaltic pump. The inlet temperature of the slurry was 35 °C as the initial temperature strongly affects the microbial inactivation (Timmermans et al., 2014). Part of the treated slurry was used for microbiological analysis, the rest of the slurry was centrifuged (20 min at 10,000 *g*) and the soluble fraction was separated. The soluble fraction was then frozen at −80 °C, freeze-dried, and stored until further analysis.

#### 2.3.2 Batch set-up

For the batch set-up, a 15 mL treatment cell was used with an electrode gap of 2 cm. The electric field strength was held constant at 10 kV (5 kV/cm) as higher values caused arcing. The actual obtained electric field strength ranged between 9.2 and 9.7 kV (4.6–4.9 kV/cm). The frequency was kept constant at 25 Hz. The energy input was varied by varying the pulse count (35/200/350/500/700 pulses). The temperature was measured directly after the treatment. Part of the slurry was used for microbiological analysis, the rest was frozen at −80 °C, freeze-dried, and stored until further analysis.

To investigate the antimicrobial effects of added compounds, 1% chitosan (Sigma-Aldrich, U.S.) was solubilised in a 1% acetic acid solution and added to the slurry at a concentration of 1%. Furthermore, for one condition the pH of the slurry was lowered to 3 with lactic acid instead of hydrochloric acid, to investigate the antimicrobial effects of organic acids. Next to this, a soluble fraction was directly PEF-treated to investigate the possible effects of shielding. All of these PEF treatments were performed with 350 pulses.

### 2.4 Microbiological analysis

To investigate the effect of pulsed electric field treatments on microbial survival, the viable number of aerobic microorganisms, anaerobic microorganisms, yeasts, and moulds were determined. For both aerobic and anaerobic microorganisms, serial dilutions were made by using 0.85% NaCl solutions and 0.1 mL was spread on plate count agar (Merck KGaA, Germany). Anaerobic conditions were created by the addition of AnaeroGen™ sachets (Oxoid; ThermoFisher Scientific, U.S.) together with the agar plates in air-tight jars. After inoculation, plates were incubated invertedly for 3 day at 30 °C. Plates that contained 10–250 colonies were counted. After counting, the plates were again incubated to detect any possible delayed growth and counted once more after one week.

For the yeast and mould counts, serial counts were made in the same way as for aerobic and anaerobic microorganisms. For diluted samples, 0.1 mL was spread on dichloran rose bengal chloramphenicol (DRBC) agar plates (Oxoid ThermoFisher Scientific, U.S.) of 100 mm × 15 mm. The agar plates contained 0.01% chloramphenicol (Oxoid ThermoFisher Scientific, U.S.) to suppress bacterial growth. For undiluted samples, 1 mL was evenly distributed over three DRBC agar plates. Plates were incubated at 25 °C for 5 days and counted after incubation. Plates that contained 10–100 colonies were counted. Yeasts and moulds were present on the same plate but were counted separately. After counting, plates were again incubated to detect any possible delayed growth and counted again after one week.

### 2.5 Protein solubility

The protein solubility was expressed as the amount of protein in the soluble fraction relative to the amount present in the initial slurry. The protein content was determined by using the Dumas method (Rapid N exceed – N/protein analyzer; Elementar, Australia). L-aspartic acid (Sigma-Aldrich, U.S.) was used as a reference. A nitrogen-to-protein conversion factor of 5.6 for soluble insect fractions and 4.76 for whole insects were used as insects also contain non-protein nitrogen compounds such as chitin (Janssen et al., 2017). The dry matter content was determined by weighing each sample before and after freeze-drying.

### 2.6 Molecular weight distribution

The protein molecular weight distribution was visualised using sodium dodecyl sulphate-polyacrylamide gel electrophoresis (SDS-PAGE). Freeze-dried material was dissolved in sample buffer (Bio-Rad Laboratories Inc., U.S.) to obtain a 2 mg/mL protein solution. The solution was mixed with Milli-Q water and β-mercaptoethanol (Sigma-Aldrich, U.S.) in a ratio of 19:20:1 for SDS-PAGE under reducing conditions. Solutions were heated for 10 min at 95 °C in a Thermomixer (Eppendorf, Germany), and centrifuged for 10 min at 14,600 *g*. For each sample, 20 μL was loaded on a 4–20% Mini-Protean TGX gel (Bio-Rad Laboratories Inc., U.S.), and 10 μL of Dual Xtra prestained protein standard (Bio-Rad Laboratories Inc., U.S.) was added to a separate lane. The electrophoresis was run at 180 V until the sample reached the bottom of the gel. After removal of the gels, they were washed with Milli-Q and stained for 1 h with Bio-safe Coomassie (Bio-Rad Laboratories Inc., U.S.). After staining, the gels were destained with Milli-Q overnight and scanned using a GS-900 calibrated densitometer (Bio-Rad Laboratories Inc., U.S.).

### 2.7 Protein secondary structure

To investigate the potential changes in secondary protein structure due to the different treatments, ATR-Fourier-transform infrared (FTIR) spectroscopy was used. Freeze-dried samples were dissolved in Milli-Q water to obtain a 5% (w/v) protein concentration. Infrared (IR) spectra were measured with an Invenio-S FTIR Spectrometer (Bruker, U.S.) using an attenuated total reflection (ATR) accessory, with a diamond-coated ZnSe crystal and a nitrogen-cooled MCT detector. The spectra were made in the range of 400–4000 cm^−1^; per measurement 68 scans were made. Milli-Q water was used as a control reference. The spectra were vector normalised and the second derivative was taken using OPUS software (Bruker, U.S.). The PEF and heat-treated (90 °C, 10 min) samples were subtracted from the untreated samples to obtain difference spectra.

### 2.8 Degree of hydrolysis

To determine the degree of hydrolysis (DH%), the percentage of free NH_2_-groups was measured using the OPA assay, based on the method described by Nielsen et al. (2001). The percentage of free NH_2_-groups was defined as the amount of measured free NH_2_-groups as a percentage of total NH_2_-groups obtained, as measured after acidic hydrolysis. Protein fractions were dissolved in Milli-Q water to obtain a 1% (w/v) protein solution. To see the increase in free NH_2_-groups, samples were measured directly after dissolution, and again after 20 h of storage at room temperature (20 °C). For the acidic hydrolysis, samples were 10x diluted in 6M HCl and kept for 24 h at 110 °C, after which they were again 10x diluted in 6M NaOH. The OPA reagent was prepared with borax (Sigma-Aldrich, U.S.), SDS (Sigma-Aldrich, U.S.), *o*-phtalaldehyde (Sigma-Aldrich, U.S.), ethanol (VWR Internation, U.S.), and DL-dithiothreitol (Sigma-Aldrich, U.S.). A calibration curve was made using L-serine (Alfa Aesar, U.S.) at concentrations of 0, 50, 100, 150, and 200 mg/L. Samples were measured at 0.01% (w/v) protein concentration in a 96-well plate (Greiner Bio-One, Germany); 20 μL of standard or sample was added and mixed with 150 μL of the OPA reagent. The absorbance at 340 nm was measured on a Multiskan FC Microplate reader (ThermoFisher Scientific, U.S.), and converted to L-serine equivalents.

### 2.9 Statistical analysis

Results were expressed as mean ± standard deviation (SD) and statistically evaluated through one-way analysis of variance (ANOVA) using SPSS 28.0.1.1 (IBM, U.S.). Statistical differences (*p* < 0.05) were analysed through Tukey tests.

## 3 Results and discussion

### 3.1 Composition of insect slurries

The dry matter, protein, and lipid content of both house cricket and lesser mealworm slurries are shown in Table 1. Lesser mealworms contain more lipids than house crickets (28.2% vs. 17.1%) as was previously shown by Sweers et al. (2023), while house crickets contain more proteins than lesser mealworms (50.5% vs. 43.8%). These differences can be explained by interspecies differences and by differences in the maturity stages of the insects (Nowak et al., 2016).

**Table 1:** Dry matter content (%), protein content (% dry weight), and lipid content (% dry weight) for house cricket and lesser mealworm slurries. Different lowercase letters in superscript indicate mean values with significant differences (p > 0.05). Statistical analyses are done separately for dry matter content, protein content, and lipid content.

Insect	Dry matter content (%)	Protein content (% dw)	Lipid content (% dw)^a^
House cricket	5.00 ± 0.69^a^	50.50 ± 1.21^a^	17.07 ± 1.00^a^
Lesser mealworm	5.24 ± 0.35^a^	43.82 ± 0.32^b^	28.18 ± 0.49^b^

a Lipid content data was first published by Sweers et al. (2023).

### 3.2 Microbial inactivation of continuous PEF processing

**Fig. 1:**
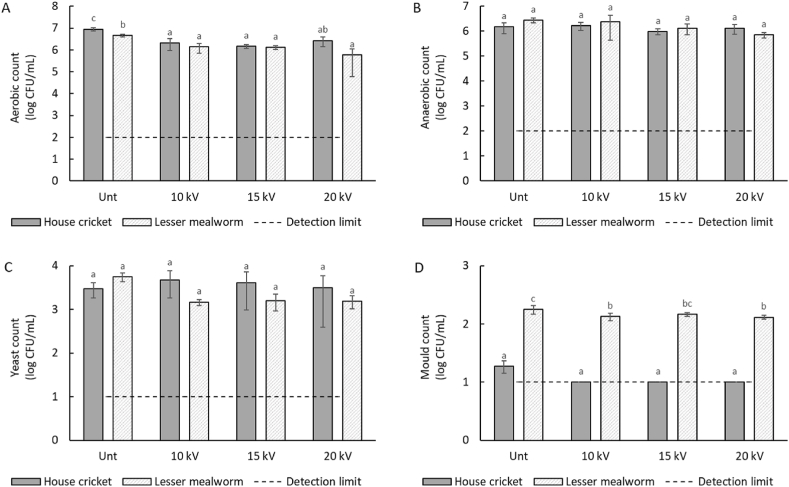
*Total aerobic (A), anaerobic (B), yeast (C), and mould (D) count of untreated (Unt) and PEF-treated (continuous set-up with 10/15/*20 kV/cm*) house cricket (HC) and lesser mealworm (LM) slurries. The detection limit indicates the lowest signal that can be observed with a sufficient degree of confidence. Different lowercase letters indicate mean values with significant differences (*p < *0.05).*

The aerobic, anaerobic, yeast, and mould counts for both house cricket and lesser mealworm slurries are shown in Fig. 1. For both house crickets and lesser mealworms, counts of aerobic and anaerobic microorganisms were similar, which could indicate a high presence of facultative anaerobic species originating from the insect gut (Tegtmeier et al., 2021). Lesser mealworms contained higher mould counts than house crickets, which was previously also found by Vandeweyer et al. (2017). Under the investigated conditions, PEF treatment decreased primarily the aerobic count for HC and LM alike. Furthermore, LM also showed a reduction in mould counts, although this was only evident with electric field strengths of 10 and 20 kV/cm. However, in all cases, even though statistically significant differences were found, these differences are all <1 log and therefore not of practical relevance. A possible reason for the minimal effect is that the energy input (20 kJ/L) was not high enough to induce practically relevant inactivation. At an electric field strength of 20 kV/cm and an energy input of 20 kJ/L, previous studies on different products also found a maximum microbial inactivation of ∼1 log for most microorganisms, although inactivation higher than 1 log was found for the Gram-negative bacteria *Salmonella Senftenberg* (∼1.5 log), the Gram-positive bacteria *Bacillus megaterium* (∼2.5 log), and the yeast *Saccharomyces cerevisiae* (∼2.5 log) (Timmermans et al., 2019; Toepfl et al., 2007). To reach higher levels of microbial inactivation, a larger energy input may be needed, but this will also increase the temperature output (Timmermans et al., 2019). The temperature increase observed in the continuous set-up in this study was <5 °C, indicating that any effect of the PEF treatment on microbial inactivation is purely caused by PEF and not by Ohmic heating. Thus, PEF treatment within the tested intensity range is not effective for microbial inactivation of insect slurries at pH 3. To check whether these intensities already affected the proteins in the insect slurries, the protein solubility and structural changes were assessed.

### 3.3 Protein solubility

**Fig. 2:**
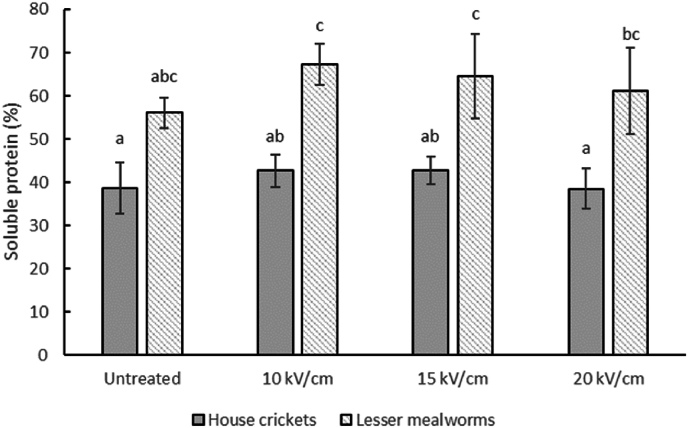
*Percentage soluble protein after continuous PEF processing at different electric field strengths (10/15/*20 kV/cm*) for house cricket and lesser mealworm slurries. Different lowercase letters in superscript indicate mean values with significant differences (*p < *0.05).*

The protein solubility after PEF processing of insect slurries in the continuous system is shown in Fig. 2. The solubility is measured by quantifying the protein content of the soluble fraction obtained after centrifugation relative to the protein content of the initial slurry. The average protein solubility is somewhat higher after PEF processing for lesser mealworms, most noticeable at 10 kV/cm. However, this is not statistically significant. A trend towards an increased protein solubility was also found by others and could be traced back to the electroporation of cell membranes, releasing more protein from the cells (Psarianos et al., 2022). In a study on mussel proteins, protein extraction also improved with a maximum extraction yield at an electric field strength of 20 kV/cm, but higher electric field strengths had a detrimental effect on protein extraction, likely caused by (thermal) protein denaturation (Zhou et al., 2017). We can generalise by saying that low-intensity PEF treatments may increase the protein extraction yield, whereas too high energy inputs decrease the protein yield.

### 3.4 Molecular weight distribution

**Fig. 3:**
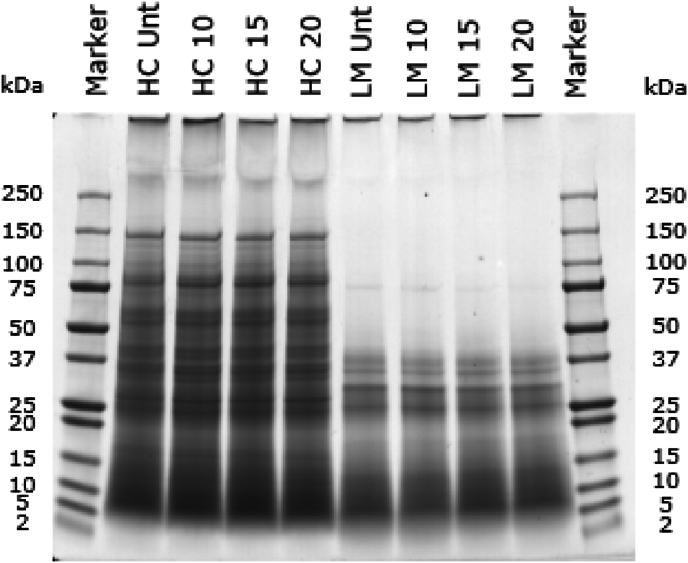
Molecular weight distribution of the proteins of house crickets (HC) and lesser mealworms (LM) measured under reducing conditions by SDS-PAGE profiles. Unt indicates untreated, and 10/15/20 indicates the electric field strength in kV/cm used during the PEF treatment with the continuous set-up.

The protein molecular weight distribution of the soluble fractions of untreated and PEF-treated slurries, as measured using SDS-PAGE under reducing conditions, is shown in Fig. 3. Clear differences are seen between the insect species. For example, unlike house crickets, lesser mealworms do not have any protein bands with a molecular weight larger than 50 kDa at the used pH of 3, except for a faint band around 75 kDa, which was previously also seen in Sweers et al. (2024b). When comparing the untreated fraction with the PEF-treated fractions, no differences are observed, indicating that the PEF treatment intensities we applied did not affect the protein molecular weight distribution. This indicates that no significant protein aggregation or dissociation occurred during PEF processing. Still, to confirm whether PEF affected the protein secondary structure, FTIR analyses were conducted.

### 3.5 Protein secondary structure

**Fig. 4:**
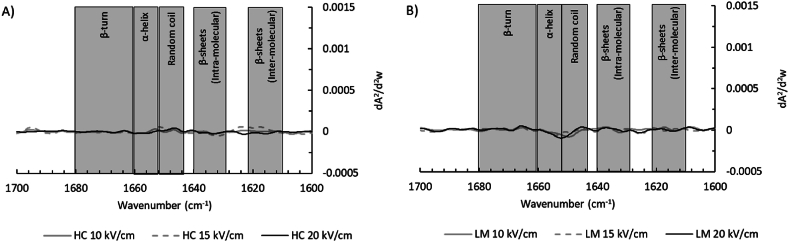
The second derivative of the amide I region of PEF-treated and heat-treated (HT) protein fractions subtracted from the untreated protein fraction for the continuous set-up (10/15/20 kV/cm) for A) soluble house cricket fractions and B) soluble lesser mealworm fractions. The spectra were obtained using Fourier-transform infrared spectroscopy (FTIR). The grey boxes are visual indicators for the protein secondary structures.

Electric fields may cause conformational changes to proteins through electrical charging and by displacement of elements by electrical forces (Bekard and Dunstan, 2013). To assess the effect of the PEF treatment on proteins, we investigated the amide I region (1700-1600 cm^−1^) through FTIR as it reveals the most significant changes in the secondary protein structure. The second derivative of the amide I region of the soluble fractions of the PEF-treated fractions subtracted from the untreated fraction can be found in Fig. 4. Again, minor differences are seen, for both house crickets and lesser mealworms. Most likely, the PEF treatment was not intense enough to induce irreversible changes in the protein conformation (Giteru et al., 2018; Takaki et al., 2021). It thus confirms that the PEF treatments also did not have any significant effect on the secondary protein structure. This is consistent with the SDS-PAGE profiles (Fig. 3), where the PEF treatments did not result in a significant effect on the protein molecular weight distribution. It would also mean that endogenous enzymes such as proteases will not be inactivated by the PEF treatment under the current intensities. To confirm this, we investigated the protease activity.

### 3.6 Hydrolysis by endogenous proteases

**Fig. 5:**
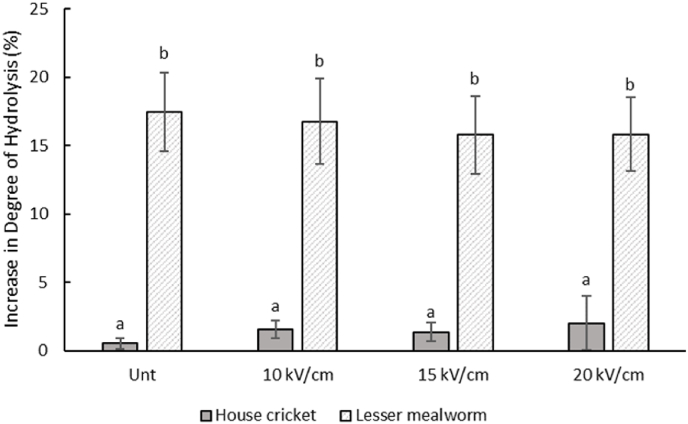
*Net increase in the percentage of free NH*_*2*_*-groups of untreated (Unt) and PEF-treated (continuous set-up with 10/15/*20 kV/cm*) soluble protein fractions from house crickets (HC) and lesser mealworms (LM) after 20 h at 20 °C and a pH value of 3. Different lowercase letters indicate significant differences (*p < *0.05).*

The endogenous proteolytic activity, indirectly measured by the increase over time at 20 °C of the number of free NH_2_-groups, can be found in Fig. 5. The value for lesser mealworms is significantly higher than for house crickets, indicating that lesser mealworms have more active proteases than house crickets at the conditions used in our study. This finding is consistent with previous literature (Sweers et al., 2024a). For both house crickets (0.5–2.0%) and lesser mealworms (15.5–17.5%), no significant differences are found between the untreated and the PEF-treated fractions. In previous literature, enzyme inactivation is obtained after PEF treatment for different food sources. This inactivation is in some cases attributed to a high temperature that is induced by the PEF treatment, and a low pH value used during processing (Akdemir Evrendilek and Hitit Özkan, 2024; Horlacher et al., 2024; Vanga et al., 2021). This confirms that PEF treatment at the current conditions did not significantly affect the protease activity. This is consistent with the PEF treatment also not significantly affecting the molecular weight distribution (Fig. 3) and the secondary protein structure (Fig. 4). For the settings used in the continuous set-up in this study, PEF is therefore not a suitable treatment to inactivate endogenous proteases.

### 3.7 PEF batch processing

#### 3.7.1 Effect of PEF batch processing on microbial inactivation

**Fig. 6:**
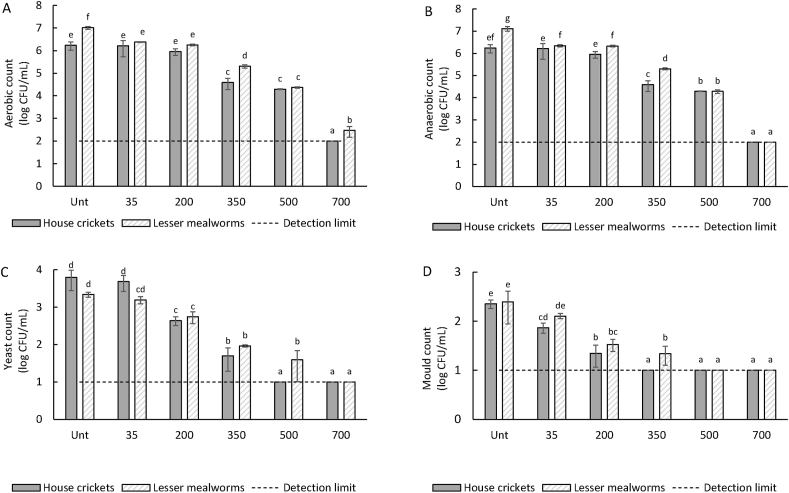
*Total aerobic (A)*, *anaerobic (B)*, *yeast (C)*, *and mould (D) counts of house cricket and lesser mealworm. Unt indicates untreated, 35/200/350/500/700 indicates the number of pulses given during the PEF treatment with batch set-up. The detection limit indicates the lowest signal that can be observed with a sufficient degree of confidence. Different lowercase letters indicate mean values with significant differences (*p < *0.05).*

To further investigate the effect of PEF on microbial inactivation of insect slurries at pH 3, a batch system was used with a fixed electric field strength of 10 kV (5 kV/cm) and a varying number of pulses (35/200/350/500/700) to obtain different levels of energy inputs. Fig. 6 shows the total aerobic, anaerobic, yeast, and mould counts. More inactivation is obtained compared to the continuous set-up: inactivation exceeds 1 log at 350 pulses, and at 700 pulses no microorganisms are detected anymore, except for aerobic microorganisms in the lesser mealworm slurry. This could be due to the higher starting concentration of aerobic microorganisms in lesser mealworms, as also seen in (Sweers et al., 2024a). The critical electric field strength, described as the electric field strength at which 50% of microorganisms survive, is said to be 10–14 kV/cm for microbial cells (Arshad et al., 2021). However, we used an electric field strength of 5 kV/cm and could still see a significant reduction. Timmermans et al. (2019) showed that in fruit juices, microbial inactivation is possible already at low electric field strengths of 0.9 and 2.7 kV/cm, but only with sufficient electrical energy input. For example, when using an electric field strength of 0.9 kV/cm (1000 μs pulse width), ∼160 kJ/kg was needed to obtain 2 log reduction of *Listeria monocytogenes*, which corresponded to a maximum temperature increase from 36 to ∼78 °C. As a comparison, when using 20 kV/cm (2 μs pulse width), the same inactivation was obtained with ∼50 kJ/kg, corresponding to a maximum temperature of ∼50 °C (Timmermans et al., 2019).

**Fig. 7:**
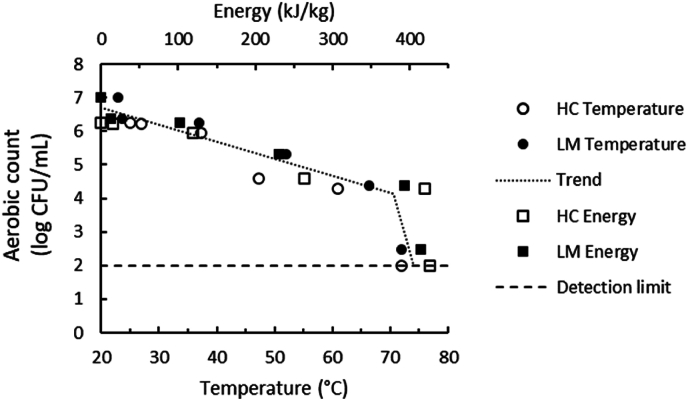
Total aerobic count of house cricket (HC) and lesser mealworm (LM) slurries plotted against the energy input (kJ/kg) that was given by the PEF device, compared with the temperature (°C) that was measured directly after the PEF treatment with batch set-up. The detection limit indicates the lowest signal that can be observed with a sufficient degree of confidence. The dotted trend line is a guide for the eye.

In Fig. 7 the count of total aerobic microorganisms is plotted against the energy input that was given by the PEF machine and the temperature that was measured directly after the PEF treatment. The inactivation is more or less linear until a turning point is reached at 500 pulses, corresponding to a temperature between 60 and 70 °C, at which the inactivation becomes significantly larger. This can be explained by the temperature of the sample reaching ∼70 °C due to Ohmic heating. The standard pasteurisation time for milk at 72 °C is 15 s, whereas the standard pasteurisation time at 62 °C is 30 min (Lewis, 2010). This increase of 10 °C thus reduces the pasteurisation time by a factor of 120. Furthermore, there is a cooperative effect between PEF and temperature, likely due to the increase in the fluidity and decrease in mechanical resistance of the cell membrane of the microorganism, making it more susceptible to electroporation (Raso et al., 2016).

The energy input in our continuous system was 20 kJ/L, and the obtained temperature increase was <5 °C. Even though the used electric field strength was higher for the continuous set-up (10–20 kV/cm) compared to the batch set-up (5 kV/cm), the obtained aerobic inactivation in the continuous set-up (<1 log) with an energy input of 20 kJ/L is consistent with the curve in Fig. 7. To reach more than 1 log microbial reduction, an energy input of at least ∼150 kJ/kg is needed. The increased energy input is thus effective in microbial inactivation, but the resulting high temperatures will also affect the protein structure.

**Fig. 8:**
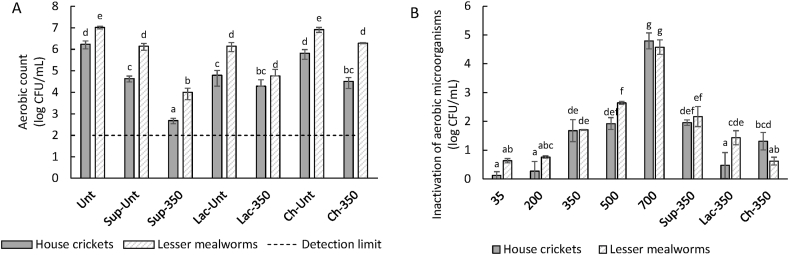
*Total aerobic counts (A) and inactivation of aerobic microorganisms (B) of house cricket and lesser mealworm slurries or supernatant (Sup). Unt indicates untreated, 35/200/350/500/700 indicates the number of pulses given during the PEF treatment with batch set-up, Lac indicates lactic acid, and Ch indicates chitosan. The detection limit indicates the lowest signal that can be observed with a sufficient degree of confidence. Different lowercase letters indicate mean values with significant differences (*p < *0.05).*

It is thus difficult to achieve sufficient microbial inactivation without the increase in temperature, which is undesired for the proteins that possess high thermal sensitivity (Zhao et al., 2012). Additional processing aids can be used in a hurdle approach. For example, Walkling-Ribeiro et al. (2011) found that a combination of PEF and microfiltration at its most effective treatment parameters (815 kJ/L) produced greater microbial inactivation and similar shelf stability compared to thermal pasteurisation (75 °C), but used a lower maximum temperature (49 °C). Furthermore, a hurdle approach consisting of PEF combined with the addition of antimicrobial compounds could be used, where antimicrobial compounds could be transported into the microorganism cells using reversible cell damage caused by electroporation (El-Kaliuoby et al., 2021; Nguyen and Mittal, 2007). We assessed the use of lactic acid and chitosan as antimicrobial hurdle compounds. As can be seen in Fig. 8A, lactic acid itself was able to reduce the number of aerobic microorganisms (1.4 log reduction for HC, 0.9 log reduction for LM), which can be explained by undissociated acids entering the bacterial cell. Chitosan addition did not have a significant effect on the number of aerobic microorganisms. The aerobic counts of the PEF-treated samples were subtracted from the aerobic counts of the untreated samples (Fig. 8B), to isolate the effect of the PEF treatment itself. There it can be seen that the addition of chitosan or lactic acid did not improve the PEF treatments at 350 pulses, and may in some cases even cause a lower effect of PEF on microbial inactivation.

**Fig. 9:**
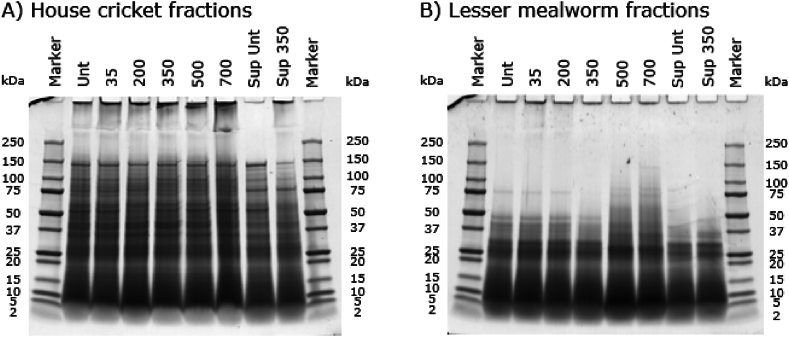
Molecular weight distribution of the proteins of (A) house cricket and (B) lesser mealworm fractions measured under reducing conditions shown by SDS-PAGE profiles. Unt indicates untreated, 35/200/350/500/700 indicates the number of pulses given during the PEF treatment with batch set-up, and sup indicates the supernatant.

Next to the effect of lactic acid and chitosan, a PEF treatment (350 pulses) on the soluble fraction instead of the complete slurry was performed to investigate any effects of shielding (Fig. 9). No statistically significant difference between a 350 pulse PEF treatment on the slurry and the supernatant is visible. The microbial counts of the supernatant were significantly lower than the microbial counts of the slurries, as during centrifugation some microorganisms end up with the pellet (D'Incecco et al., 2023). The results thus suggest that combined centrifugation and PEF treatment could form a hurdle approach to obtain a sufficient degree of inactivation with a relatively mild PEF treatment, as this combination causes a 3.3 log reduction for house crickets and a 3.0 log reduction for lesser mealworms.

#### 3.7.2 Effect of PEF batch processing on protein structure and molecular weight distribution

Fig. 9 shows the protein molecular weight distributions of the untreated and PEF-treated fractions, as measured using SDS-PAGE under reducing conditions. In lanes 2–7, the slurries were measured instead of just the soluble fractions, which may explain differences with Fig. 3. For house cricket fractions, no clear differences are seen between untreated and PEF-treated fractions. For the lesser mealworm fractions, differences can be seen between the untreated fractions and the more intense PEF treatments of 500 and 700 pulses. For untreated fractions and less intense PEF treatments (350 pulses and lower) no clear protein bands larger than 50 kDa were observed, while PEF treatments of 500 and 700 pulses show clear protein bands between 50 and 100 kDa. It could be that at these higher intensities, some protein aggregation takes place, which could be caused by the temperature increase. The relatively high conductivities at 35 °C of the lesser mealworm slurry (3.5 mS/cm) and house cricket slurry (4.4 mS/cm) will have led to more Ohmic heating compared to lower conductivities. Therefore, pH 3, although having benefits such as high protein solubility and inhibition of enzymatic browning, was one of the causes of the increased conductivity by the addition of more salt.

**Fig. 10:**
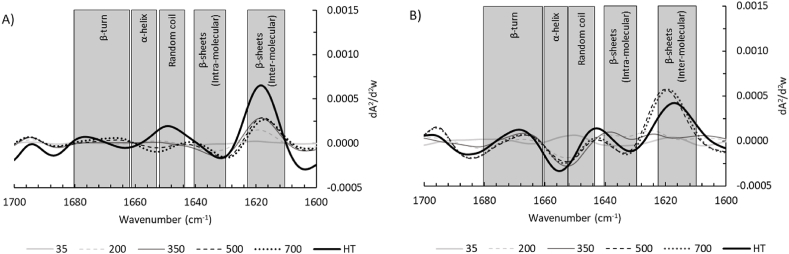
The second derivative of the amide I region of PEF-treated and heat-treated (HT) protein fractions subtracted from the untreated protein fraction for the batch set-up (35/200/350/500/700 pulses) for A) soluble house cricket fractions and B) soluble lesser mealworm fractions. The spectra were obtained using Fourier-transform infrared spectroscopy (FTIR). The grey boxes are visual indicators for the protein secondary structures.

Fig. 10 shows the second derivatives of the amide I region (1700-1600 cm^−1^) of the heat- and PEF-treated (batch set-up) fractions, with the values of the untreated fractions subtracted. Already at 200 pulses the proteins of house crickets show more inter-molecular β-sheets, whereas lesser mealworm proteins show the same starting from 500 pulses. The lesser mealworm proteins being affected at 500 and 700 pulses is consistent with the SDS-PAGE profiles (Fig. 9B). The higher lipid content in the lesser mealworm slurry may shield the proteins from the PEF treatment, which was previously demonstrated for bacterial spores in milk (Bermúdez-Aguirre et al., 2012).

A higher prevalence of inter-molecular β-sheets (around 1620 cm^−1^) can indicate protein aggregation and denaturation, which can lead to lower protein solubility (Shivu et al., 2013). This suggests that at higher energy inputs, as used in this batch set-up, the protein solubility might in fact decrease instead of the slight increase that was seen in Fig. 2. This would be in line with earlier observations for mussel proteins (Zhou et al., 2017). For house crickets, no further increase in inter-molecular β-sheets was seen between 350 and 700 pulses, whereas heating led to a significant additional increase in inter-molecular β-sheets. For lesser mealworms, 35–350 pulses do not significantly impact the inter-molecular β-sheets as mentioned earlier, whereas 500 and 700 pulses do cause a significant increase in inter-molecular β-sheet levels. Furthermore, it was observed that for heat treatment, the peak maximum was at a slightly lower frequency band position (1617 cm^−1^) compared to PEF treatments with 500 and 700 pulses (1619-1620 cm^−1^), which is typical for aggregation processes involving intermolecular interactions (Shivu et al., 2013). For house crickets, the peak maximum of the heat treatment (1618.5 cm^−1^) was similar to the peak maximum of PEF treatments (1617-1620 cm^−1^).

For house crickets, less obvious effects are found for other typical secondary protein structures. The effects of different PEF intensities on intra-molecular β-sheets are similar to the previously described effects on inter-molecular β-sheets, although the effect is less and instead of an increase there is a decrease. Heating increased the level of random coil structures, whereas the PEF treatments did not affect the prevalence of random coils. For lesser mealworms, stronger effects are found for other secondary protein structures. The intra-molecular β-sheet levels decreased for the heat treatment and the more severe PEF treatments (500 and 700 pulses). The changes in protein structure at the 500 and 700 pulses PEF treatment were also seen in band patterns on the SDS-PAGE gel (Fig. 9B) and the previously discussed inter-molecular β-sheet levels. Both PEF treatments (>200 pulses) and the heat treatment reduced the amount of α-helices, especially after heating. This was previously seen for PEF treatments (5–25 kV) of egg white protein powder, but these treatments used a much higher pulse count of 45,000 (Qian et al., 2016). Thus, for our results, it cannot be concluded whether the effects of the PEF treatments are due to PEF itself or due to the Ohmic heating.

Although PEF treatments with higher energy inputs were successful in microbial inactivation, this goes together with a temperature increase and a loss in native protein structure. Furthermore, even though PEF treatments in continuous systems are more uniform than in batch systems (Wouters et al., 1999), it may be challenging to scale up PEF treatments to a larger-scale continuous system, as results are highly dependent on the exact PEF processing parameters and production conditions (Jin et al., 2015). To conclude, PEF-induced microbial inactivation with inactivation values that are relevant for industry is possible, but PEF cannot be considered to be a non-thermal decontamination treatment, as the practically relevant decreases in microbial load cannot be achieved without the PEF-induced temperature increase.

## 4 Conclusion

We investigated the effect of pulsed electric field (PEF) processing on microbial inactivation and modifications to the native protein structure at a pH value of 3 for house crickets and lesser mealworms. Continuous PEF treatment at ∼20 kJ/L significantly reduced the microbial load, but this reduction in microbial load was not of practical relevance (<1 log). Higher reductions in microbial load up to 5 log were achieved with higher energy inputs (>150 kJ/kg) using batch PEF treatment but caused increased temperatures up to 75 °C, which affected the protein structure, as was seen in SDS-PAGE chromatograms and FTIR spectra. The lower-intensity continuous PEF treatments did not cause noteworthy temperature increases (<5 °C) and did not affect the protein structure and proteolytic activity. We conclude that PEF processing for successful microbial inactivation cannot be considered a non-thermal treatment under the applied conditions. Nonetheless, PEF may be suitable for microbial inactivation when combined with other techniques (i.e. hurdle approach), such as combinations of PEF with moderate heat, microfiltration, or centrifugation, which may be milder on proteins compared to conventional thermal treatment. Further studies are needed to understand the applicability of this approach in other PEF set-ups. In addition, future research should investigate the synergistic effects of PEF treatment and other mild processing methods for effective microbial inactivation while preserving native protein structure. Furthermore, the possibility of using optimal PEF intensities to increase the protein solubility in lesser mealworms should be investigated.

## CRediT authorship contribution statement

**L.J.H. Sweers:** Conceptualization, Methodology, Formal analysis, Investigation, Writing – original draft. **M. Mishyna:** Conceptualization, Writing – review & editing, Supervision. **L.M. Ahrné:** Conceptualization, Writing – review & editing. **R.M. Boom:** Conceptualization, Writing – review & editing, Supervision. **V. Fogliano:** Conceptualization, Writing – review & editing, Supervision. **T. Patra:** Conceptualization, Writing – review & editing. **C.M.M. Lakemond:** Conceptualization, Writing – review & editing, Supervision. **J.K. Keppler:** Conceptualization, Writing – review & editing, Supervision.

## Declaration of competing interest

The authors declare that they have no known competing financial interests or personal relationships that could have appeared to influence the work reported in this paper.

## Data Availability

Data will be made available on request.
